# Oxygenation index and NT-proBNP as predictors of pulmonary hypertension and ventilation/perfusion mismatch in acute pulmonary embolism

**DOI:** 10.3389/fcvm.2023.1090805

**Published:** 2023-02-06

**Authors:** Wenjing Ye, Xi Chen, Xiaoming Li, Xuejun Guo, Wen Gu

**Affiliations:** Department of Respiratory Medicine, Xinhua Hospital, School of Medicine, Shanghai Jiao Tong University, Shanghai, China

**Keywords:** pulmonary embolism, biomarkers, pulmonary artery pressure, V/Q mismatch, linear regression

## Abstract

**Introduction:**

The magnitude of pulmonary artery pressure (PAP) and the extent of ventilation/perfusion (V/Q) mismatch are essential for assessing the prognosis of acute pulmonary embolism (APE). We aimed to develop a model for predicting the status of the pulmonary circulation and arterial gas exchange functions using serum levels of cardiac biomarkers and arterial oxygenation index (OI) values.

**Materials and methods:**

This single-center, retrospective observational cohort study included 224 patients with APE. Multivariate linear regression and Poisson regression were used to test the statistical association between cardiac biomarkers, OI, PAP, and V/Q mismatch. Diagnostic efficiency was calculated from a receiver operating characteristic (ROC) curve.

**Results:**

Serum levels of troponin I (TNI), N-terminal pro-brain natriuretic peptide (NT-proBNP), and arterial OI magnitude significantly correlated with PAP and V/Q mismatches (*P* < 0.05). Multivariate linear regression showed that NT-proBNP serum levels (β = 0.002, *P* < 0.001) and OI values (β = −0.022, *P* = 0.001) significantly influenced PAP. Arterial OI (β = −0.039, *P* < 0.001) had a significant influence on the percentage of pulmonary vascular obstruction (PVO) as determined by perfusion scanning. Poisson regression showed that OI (odds ratio: 0.995, *p* < 0.001) was a predictor of the number of lung segments with V/Q mismatches. ROC area under the curve (AUC) values of NT-proBNP and OI predicting pulmonary hypertension were 0.716 and 0.730, respectively, and for V/Q mismatch scanning, the results were 0.601 and 0.634, respectively.

**Conclusion:**

Arterial OI and serum levels of cardiac biomarkers may be used as indicators of pulmonary hypertension and V/Q mismatch.

## 1. Introduction

Venous thromboembolism (VTE), including acute pulmonary embolism (APE) and deep vein thrombosis, is the third most common acute cardiovascular disease in the world (1). Longitudinal studies have found that the annual incidence of APE shows an upward trend over time (2, 3). Precise diagnosis and early determination of risk stratification in patients with APE are critical for follow-up treatment.

Both pulmonary circulation and arterial gas exchange are affected by APE. The main manifestations of disrupted pulmonary circulatory are increased pulmonary artery pressure (PAP) and right ventricular (RV) failure (4). Persistent and organized deep vein thrombosis can result in a progressive increase in PAP that can eventually cause chronic thromboembolic pulmonary hypertension (CTEPH), a life-threatening obstructive vasculopathy. A mean PAP of >30 mmHg has been related to poor survival (4). Therefore, pre-existing CTEPH or high PAP should not be overlooked in patients with APE (1).

Abnormal arterial gas exchange is caused by reduced blood flow at the APE site and the widening of alveolar dead space. Ventilation/perfusion (V/Q) mismatch will ultimately result in respiratory failure (5). V/Q scintigraphy is a diagnostic test for APE, especially for finding segmental V/Q mismatches.

The extent of V/Q mismatch at diagnosis is the main predictor of poor APE prognosis (6). In addition to pulmonary circulation function, risk stratification and adverse outcomes in APE have also been increasingly studied in relation to respiratory perfusion functions such as partial pressure of oxygen (PO_2_) and oxygenation index (OI) (7, 8).

For patients initially admitted with APE, it is often easier to measure serum levels of cardiac biomarkers and arterial blood gas values than conduct echocardiography and V/Q scans. Given the importance of PAP and V/Q for prognosis, our research aimed to evaluate the use of laboratory results to predict the status of the pulmonary circulation and arterial gas exchange functions.

## 2. Materials and methods

### 2.1. Study design and participants

From January 2018 to April 2021, patients meeting the criteria for the diagnosis, treatment, and prevention of pulmonary thromboembolism (Chinese version) (9) at the Department of Respiratory Medicine, Xin Hua Hospital, affiliated with the Shanghai Jiao Tong University School of Medicine, were enrolled in this study. The exclusion criteria were: (1) age < 18 years, (2) PE without verification (3) pulmonary hypertension (PH) caused by severe cardiovascular or other diseases, and (4) other lung diseases (chronic obstructive pulmonary diseases, asthma, pneumonia, etc.). The risk stratification approach used in our study was also based on Chinese guidelines (Supplementary Table 1; 9).

### 2.2. Ethical approval

The study was approved by the Medical Ethics Committee of Xinhua Hospital, affiliated with Shanghai Jiao Tong University School of Medicine, Shanghai, China (No. XHEC-NSFC-2020-014) and the informed consent was exempted from all participants.

### 2.3. Data collection

General information about the participants was collected, including sex and age. Laboratory examination data included arterial blood gas measurement, serum levels of cardiac biomarkers troponin I (TNI) and N-terminal pro-brain natriuretic peptide (NT-proBNP), and D-dimer values. The arterial blood gas and cardiac biomarkers were collected at the time of admission, usually within one hour.

### 2.4. Ventilation-perfusion lung scintigraphy

High probability findings of V/Q scan include at least two large mismatched segmental defects or segmental defect equivalents (10, 11). The diagnosis of APE was confirmed by combining a high probability of V/Q scan mismatch with clinical data. The number of lung segments with V/Q mismatches was collected for each patient. To semi-quantitatively study the mismatch range, each V/Q scan was scored as follows (12). According to the regional distribution of pulmonary blood flow in the supine position, each lobe was assigned a weight: right upper lobe 18%, right middle lobe 12%, right lower lobe 25%, left upper lobe 13%, left lingula 12%, and left lower lobe 20%. For comparison with the photo density of a normally perfused area, a semi-quantitative perfusion fraction for each lobe from 0 to 1 (0, 0.25, 0.5, 0.75, 1) was evaluated. Each lobe perfusion score was calculated by multiplying the perfusion fraction by the corresponding weight. The percentage of pulmonary vascular obstruction (PVO) was calculated as: (PVO) = (1–Overall perfusion score) × 100.

For low-risk and intermediate-risk patients, the V/Q scan was completed about 3 days after admission. For high-risk patients, especially high-risk patients, the V/Q scan was taken after patients had stabilized, usually within 5 days after admission.

### 2.5. Echocardiography

APE may cause RV dysfunction, which can be observed by echocardiography (12). Pulmonary artery systolic pressure can be determined by measuring the tricuspid regurgitation pressure difference and the right atrial pressure using echocardiography. We collected the PAP data of each patient in this study. The detection time of echocardiography was the same as that of V/Q scan.

### 2.6. Statistical analysis

SPSS 19.0 (IBM Corp., Armonk, NY, USA) and R software (version 4.2.1) were used in this study. The normality of the data distribution was checked using the Kolmogorov-Smirnov test. Continuous data are summarized as mean with standard deviation (SD) or median with interquartile range (IQR). The non-parametric Mann–Whitney test was used to analyse the non-normally distributed data. ANOVA test was used to analyse normally distributed data between groups, and LSD was used in differences between couples of groups. Categorical data were summarized as percentages and fractions of occurrence and were analysed by chi-square test to compare groups according to the variable type. The correlation between the variables was measured using Spearman’s correlation coefficient. Generalized and multivariate linear regression were used to explore the factors affecting PH and the extent of lung segment V/Q mismatches. We used Poisson regression when the outcome variable was a count-type variable. The receiver operating characteristic (ROC) curve and area under the curve (AUC) were used to determine the diagnostic value and optimal cut-off values. *P*-values were two-sided, and <0.05 was considered significant.

## 3. Results

### 3.1. Patient characteristics

Overall, 224 patients met the inclusion criteria. Patient numbers in the high, intermediate, and low-risk groups were 31 (13.8%), 97 (43.3%), and 96 (42.9%), respectively. A total of 89 patients received oxygen therapy, 19 (61.3%) high-risk patients, 42 (43.3%) intermediate-risk patients, and 28 (29.2%) low-risk patients. The values of D-dimer, TNI, NT-proBNP, PO_2_, OI, PAP, number of lung segments with V/Q scan mismatches, and PVO between the different risk stratification groups were statistically significant (Table 1). Differences between couples of groups were shown in Supplementary Table 2.

**TABLE 1:** Acute pulmonary embolism (APE) patient characteristics.

	Total *N* = 224	High-risk *N* = 31	Intermediate-risk *N* = 97	Low-risk *N* = 96	*P*
Male gender (%)	103 (32.3%)	10 (32.3%)	43 (44.3%)	50 (52.1%)	0.143
Age (years)	68.44 (13.48)	70.87 (12.91)	68.04 (13.15)	68.06 (14.04)	0.56
D-Dimer (mg/L)	1.64 (0.70–3.51)	2.31 (0.92–5.39)	2.38 (0.99–3.84)	1.09 (0.39–2.74)	<0.001
TNI (ng/ml)	0.01 (0.01–0.04)	0.03 (0.01–0.06)	0.04 (0.01–0.08)	0.01 (0.00–0.01)	<0.001
NT-proBNP (pg/ml)	230.25 (80.17–1037.78)	847.00 (234.30–2450.00)	729.70 (271.15–2344.50)	77.71 (30.73–166.45)	<0.001
PO_2_ (kpa)	13.16 (4.58)	10.87 (2.87)	12.76 (4.55)	14.30 (4.74)	0.001
OI (mmHg)	374.18 (105.64)	281.08 (88.40)	361.83 (104.39)	416.72 (88.91)	<0.001
PAP (mmHg)	30 (25–36)	35 (26–44)	35 (28.5–41.5)	26.5 (23–31)	<0.001
Number of V/Q mismatched lung segments (*n*)	2.5 (0–6)	16 (0–16)	2 (0–5)	2 (0–4)	<0.001
PVO (%)	17.60 (0.00–31.65)	41.70 (37.57–48.71)	17.45 (0.00–25.65)	12.91 (0.00–23.02)	<0.001
Hospitalization days (range)	12 (10–14)	12 (10–15)	12 (10–15)	11 (9–14)	0.025

Data are presented as mean (SD), median (IQR), *n* (%). TNI, troponin I; NT-proBNP, N-terminal pro-brain natriuretic peptide; PO_2_, partial oxygen pressure; V/Q scan, ventilation-perfusion lung scintigraphy; PAP, pulmonary artery pressure; PVO, pulmonary vascular obstruction; OI, oxygenation index.

### 3.2. Correlation between cardiac Biomarkers, OI, PH, and PVO

OI is not affected by the oxygen absorption concentration, and it can better reflect patient hypoxia than the partial oxygen pressure measured in arterial blood. Therefore, we chose OI for correlation analysis. TNI, NT-proBNP, and OI were significantly correlated with systolic PAP and pulmonary perfusion defects (*P* < 0.05) (Table 2). NT-proBNP and OI were more significantly correlated than TNI.

**TABLE 2:** Correlation between cardiac biomarkers, oxygenation index (OI), pulmonary hypertension (PH), and pulmonary vascular obstruction (PVO).

	PAP (mmHg)	Number of lung segments with mismatched V/Q scan (*n*)	PVO (%)
TNI (ng/ml), (SCC, *P*)	0.368, *P* = 0.000	0.135, *P* = 0.043	0.149, *P* = 0.026
NT-proBNP (pg/ml), (SCC, *P*)	0.377, *P* = 0.000	0.212, *P* = 0.001	0.223, *P* = 0.001
OI (mmHg), (SCC, *P*)	−0.275, *P* = 0.000	−0.343, *P* = 0.000	−0.297, *P* = 0.000

SCC, Spearman Correlation Coefficient; TNI, troponin I; NT-proBNP, N-terminal pro-brain natriuretic peptide; V/Q scan, ventilation-perfusion lung scintigraphy; PAP, pulmonary artery pressure; PH, pulmonary hypertension; PVO, pulmonary vascular obstruction.

### 3.3. Univariate and generalized multivariate linear regression models

**FIGURE 1:**
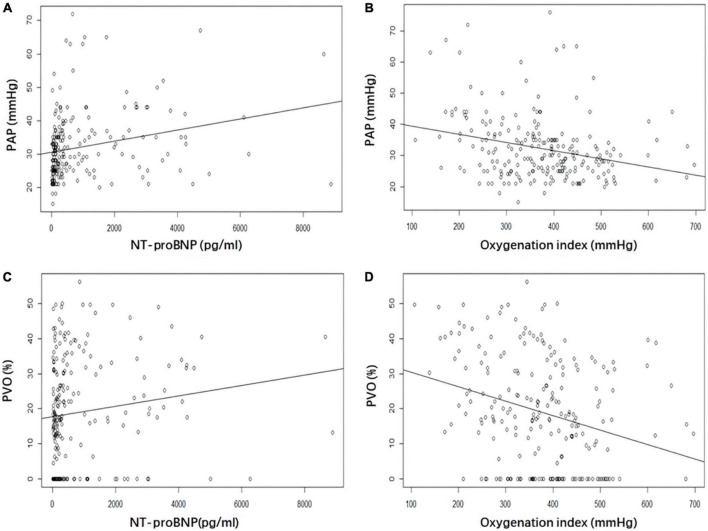
Correlation between NT-proBNP and oxygenation index (OI) with pulmonary artery pressure (PAP) and pulmonary vascular obstruction (PVO). **(A)** NT-proBNP and PAP. **(B)** OI and PAP. **(C)** NT-proBNP and PVO. **(D)** OI and PVO. NT-proBNP, N-terminal pro-brain natriuretic peptide; PAP, pulmonary artery pressure; PVO, pulmonary vascular obstruction; OI, oxygenation index.

In the univariate models, significant correlations were evident between NT-proBNP, OI, PAP, and PVO (Figure 1). Multivariate linear regression analysis showed that NT-proBNP (β coefficient = 0.002, *P* < 0.001) and OI (β coefficient = −0.022, *P* = 0.001) significantly affected PAP. The OI (β coefficient = −0.039, *P* < 0.001) also significantly influenced PVO. PAP and PVO decreased by 2.2 mmHg and 3.9%, respectively, per 100 mmHg increase in the OI. The NT-proBNP level did not significantly influence PVO (*P* = 0.279). No significant association was found between TNI and PAP, PVO in both univariate and multivariate Models (Table 3).

**TABLE 3:** Influencing factors by linear regression analysis.

	Univariate linear regression		Multivariate linear regression	
	β Coefficient (95% CI)	*P*	β Coefficient (95% CI)	*P*
**Factor for PAP**				
NT-proBNP (pg/ml)	0.002 (0.000,0.003)	<0.001	0.002 (0.001,0.003)	<0.001
OI (mmHg)	−0.026 (−0.038, −0.014)	<0.001	−0.022 (−0.034, −0.010)	0.001
TNI (ng/ml)	10.133 (−1.907, 22.175)	0.09	−0.402 (−4.403, 3.599)	0.843
**Factor for PVO**				
NT-proBNP (pg/ml)	0.001 (0.000, 0.002)	0.045	0.001 (0.000, 0.002)	0.279
OI (mmHg)	−0.041 (−0.060, −0.023)	<0.001	−0.039 (−0.058, −0.020)	<0.001
TNI (ng/ml)	13.290 (−5.465, 32.046)	0.164	3.223 (−3.038, 9.484)	0.952

TNI, troponin I; NT-proBNP, N-terminal pro-brain natriuretic peptide; PAP, pulmonary artery pressure; PVO, pulmonary vascular obstruction; OI, oxygenation index.

Table 4 shows the Poisson regression analysis used to identify the predictors of the number of lung segments with V/Q scan mismatches and hospitalization days. Odds ratios (OR) with 95% confidence intervals (CI) were also calculated. OI was a significant variable to identify the predictors of the number of lung segments with V/Q scan mismatches (OR 0.995; 95% CI: 0.995–0.996; *P* < 0.001). NT-proBNP (OR 1.001; 95% CI: 1.000–1.003; *P* = 0.034), OI (OR 0.999; 95% CI: 0.998–1.000; *P* = 0.043) and PAP (OR 1.006; 95% CI: 1.003–1.008; *P* = 0.020) were significant variables to identify the predictors of hospitalization time.

**TABLE 4:** Poisson regression for predictors of the number of V/Q mismatched lung segments and hospitalization time.

	Odds ratio	95% Confidence interval	*P*
**Number of V/Q mismatched lung segments**			
NT-proBNP (pg/ml)	1.000	0.999–1.000	0.295
OI (mmHg)	0.995	0.994–0.996	<0.001
TNI (ng/ml)	1.261	0.715–2.126	0.403
**Hospitalization days**			
NT-proBNP (pg/ml)	1.001	1.000–1.003	0.034
OI (mmHg)	0.999	0.998–1.000	0.043
TNI (ng/ml)	1.156	0.931–1.435	0.505
PAP (mmHg)	1.006	1.003–1.008	0.020
PVO (%)	1.001	0.999–1.003	0.490

TNI, troponin I; NT-proBNP, N-terminal pro-brain natriuretic peptide; OI, oxygenation index; PAP, pulmonary artery pressure; PVO, pulmonary vascular obstruction.

### 3.4. Diagnostic accuracy of parameters for PH and the extent of lung segment defect

PH was considered to exist if the pulmonary artery systolic pressure obtained by echocardiography was greater than 30 mm Hg. At least two large mismatched segmental defects were considered positive for the V/Q scan results.

The ROC analysis showed that NT-proBNP (AUC 0.716, 95% CI: 0.645–0.787, *P* < 0.001) and OI (AUC 0.730, 95% CI: 0.650–0.810, *P* < 0.001) had positive effects in predicting PH. The optimal cut-off values for each parameter were as follows: NT-proBNP: 328.85 pg/ml (sensitivity 80.00%; specificity 62.28%); OI: 372.86 mmHg (sensitivity, 62.28%; specificity, 81.82%).

**FIGURE 2:**
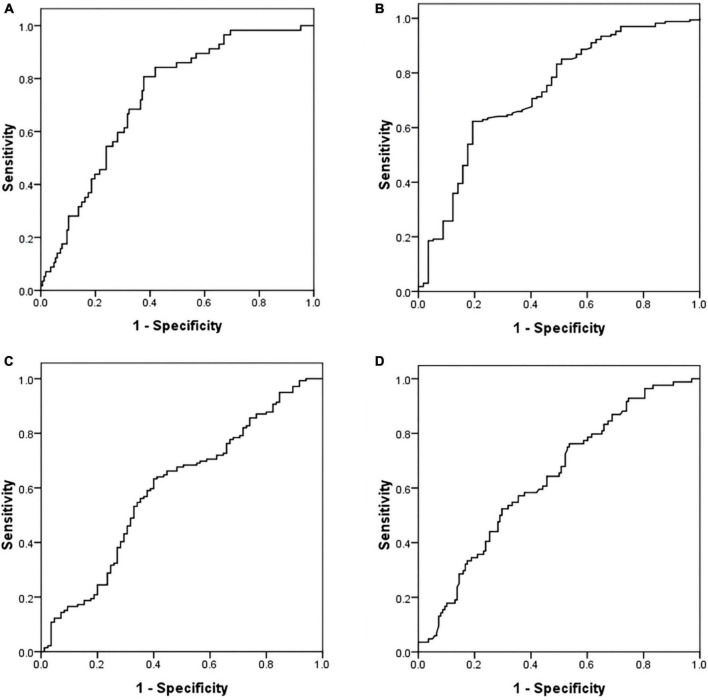
Receiver operating characteristic (ROC) curve of NT-proBNP and oxygenation index (OI) in predicting PH and V/Q scan result. **(A)** NT-proBNP and PH. **(B)** OI and PH. **(C)** NT-proBNP and V/Q scan result. **(D)** OI and V/Q scan result. ROC, receiver operating characteristic; NT-proBNP, N-terminal pro-brain natriuretic peptide; PH, pulmonary hypertension; V/Q, ventilation perfusion lung scintigraphy; OI, oxygenation index.

The AUC of NT-proBNP and OI were 0.601 (95% CI: 0.523–0.678, *P* = 0.012) and 0.634 (95% CI: 0.561–0.708, *P* = 0.038). They had a predictive value for the V/Q scan results. The critical values of NT-proBNP and OI were 211.90 pg/ml (sensitivity, 63.04%; specificity, 60.71%) and 406.59 mmHg (sensitivity, 52.38%; specificity, 70.29%) (Figure 2).

## 4. Discussion

In 2001, the Respiratory Branch of the Chinese Medical Association issued the first guidelines for standardized diagnosis and treatment of pulmonary embolism (9). The prevention and treatment of APE and VTE in China have undergone tremendous changes with the help of joint efforts. The mortality rate of pulmonary embolism in China has dropped from 25% 20 years ago to 3.9% (13), and the latest data show a further drop to 1.3% (14). The change in this trend was associated with successful adherence to the risk-adjusted diagnostic and treatment plans recommended by the guidelines (9).

A tricuspid valve insufficiency gradient > 30 mmHg represents the RV overload and dysfunction criterion and is seen in over 30% of patients with pulmonary embolism (15). A long-term study over 15 years found that mortality correlated with PAP and RV failure. PH progressed further in patients with an initial PAP > 30 mmHg. In contrast, almost none of the patients with a normal initial PAP developed severe PH (16). Therefore, it is crucial to assess PAP at the time of initial APE onset. At present, echocardiography is not recommended for patients with suspected PE yet stable hemodynamics (10). Furthermore, not all hospitals can perform primary echocardiography; hence, we wanted to develop a model to predict PAP changes quickly through simple and rapid laboratory examinations.

A lung perfusion mismatch in a lobar, segmental, or even sub-segmental distribution and without a ventilation defect can be observed on a V/Q scan (17). V/Q scanning is the standard for assessing PVO (18) that can evaluate the extent and degree of embolism. During an 8-year follow-up study, PVO at the initial PE diagnosis (HR: 33.00, 95% CI: 1.64–667.00, *P* = 0.02) was the main predictor for CTEPH (6). A V/Q scan appointment always involves long wait times, is not as fast as the computed tomography pulmonary angiogram (CTPA) test, and is not available at all hospitals. However, given its significant prognostic value, the establishment of PVO prediction models will help to rapidly evaluate the condition of APE patients and guide the follow-up treatment plan.

Our team has been committed to the evaluation and prognosis of APE. A V/Q defect ultimately results in respiratory failure and hypoxemia. However, according to Chinese guidelines (9), risk stratification mainly depends on the patient’s hemodynamics, cardiac biomarkers, and RV function and omits the role of respiratory function. Hypoxemia is a typical manifestation of APE (19). Our previous studies have found that PO_2_ and OI are prognostic biomarkers of mortality in APE. Low PO_2_ (less than 8 kPa) suggested a higher risk of mortality (HR 9.462, *P* = 0.001) (7). We designed this study to elucidate the importance of respiratory function further and improve clinical focus. We found that the OI significantly influenced both PAP and PVO. This suggests that the OI has strong clinical value for assessing pulmonary circulation and arterial gas exchange in APE patients.

Serum NT-proBNP level reflects the status of hemodynamics and RV function in APE patients. A high NT-proBNP level (more than 500 pg/ml) was used to select patients for outpatient treatment in a multicenter study (20). Focusing on the prognostic specificity of adverse outcomes, an NT-proBNP value of ≥600 pg/ml is more appropriate (21). In our study, the optimal cut-off value of NT-proBNP in patients with high PAP (>30 mmHg) was 328.85 pg/ml. PAP increased by 1 mmHg per 500 pg/ml increase in NT-proBNP level. The decrease in RV contractile forces causes an imbalance in oxygen supply and demand, and the two can interact. Although NT-proBNP level was associated with PVO through simple linear regression, it was not statistically significant. This might suggest a limitation of NT-proBNP, different from the OI, in assessing gas exchange in APE.

Our study found that TNI correlated with PAP and PVO, but linear regression analysis suggested that it had no predictive value for either. Pulmonary artery obstruction and hypoxic vasoconstriction cause RV dysfunction and increased pulmonary vascular resistance in APE patients (22). Unlike acute coronary syndrome, coronary artery occlusion or spasm causes myocardial ischemia and necrosis (23). TNI is a marker of myocardial injury but not of RV dysfunction. This may explain why TNI was not an effective predictor of PAP.

Our study has some limitations. First, the sample size was small, and it was a single-center retrospective study. The patients included in our study were all hospitalized; therefore, they were mainly in intermediate- and high-risk groups. The enrolled patients might have overshadowed low-risk patients who only required outpatient treatment and follow-up. The patients who were haemodynamically unstable and unable to complete the echocardiography and V/Q scan were not included in this study. We conducted the linear regression between different risk groups of APE, but there was no significant statistical significance in the subsequent analyses (Supplementary Table 3), which might due to the shortage and deviation of sample size. Second, although the V/Q scan is an effective imaging method for detecting pulmonary embolism, it is a planar image, which has certain limitations in measuring the specific perfusion defect range of the patient. Although we measured PVO artificially, it could only be detected semi-quantitatively. Third, the gold standard for the diagnosis of PH is the RV floating catheter measurement. Almost no patients underwent this invasive examination; therefore, we used PAP measured by echocardiography for the research analysis. Finally, we need to expand the patient sample size further and collect V/Q single photon emission/computed tomography (SPECT/CT) data (1, 24) that can be used for three-dimensional imaging, to verify the applicability of the results.

In conclusion, our study found that PAP and PVO decreased by 2.2 mmHg and 3.9%, respectively, per 100 mmHg increase in the OI, and PAP increased by 1 mmHg per 500 pg/ml increase in the NT-proBNP. OI has strong clinical value for assessing the pulmonary circulation and arterial gas exchange of APE patients. This study provides a potentially convenient method for clinical evaluation of APE when it is challenging to perform immediate echocardiography and V/Q scans.

## Data availability statement

The original contributions presented in this study are included in this article/Supplementary material, further inquiries can be directed to the corresponding authors.

## Ethics statement

The studies involving human participants were reviewed and approved by Medical Ethics Committee of Xinhua Hospital, affiliated with Shanghai Jiao Tong University School of Medicine, Shanghai, China. Written informed consent for participation was not required for this study in accordance with the national legislation and the institutional requirements.

## Author contributions

WY and XC collected the data. WY and XL performed the data analysis. WY, XC, XG, and WG wrote the main manuscript. WY, XC, and XL prepared the Figures 1, 2 and Tables 1–4. XG and WG designed the study, revised, and reviewed the manuscript. All authors reviewed the manuscript.
