# Supplementary material for: Oxygenation index and NT-proBNP as predictors of pulmonary hypertension and ventilation/perfusion mismatch in acute pulmonary embolism
Source: Front Cardiovasc Med. 2023 Feb 6;10:1090805. doi: 10.3389/fcvm.2023.1090805 (PMC9940751; doi:10.3389/fcvm.2023.1090805)
Supplement: Supplementary file 1 [file Table_1.docx]

STable 1. Risk stratification of patients with pulmonary embolism according to the Chinese guidelines

| Risk stratification | | Shock or hypotension | Sign of RVD in imaging test^a^ | Elevated lab cardiac biomarkers^b^ |
| --- | --- | --- | --- | --- |
| High risk | | + | + | +/- |
| Intermediate risk | Intermediate-high risk | - | + | + |
|  | Intermediate-low risk | - | +/-^c^ | -/+ ^c^ |
| Low risk | | - | - | - |

NOTE: RVD: right heart dysfunction; a: Echocardiographic criteria of RV dysfunction include RV dilation and/or an increased end-diastolic RV–LV diameter ratio (in most studies, the reported threshold value was 0.9 or 1.0); hypokinesia of the free RV wall; increased velocity of the tricuspid regurgitation jet; or combinations of the above. On computed tomographic (CT) angiography (four-chamber views of the heart), RVD is defined as an increased end-diastolic RV/LV (left ventricular) diameter ratio (with a threshold of 0.9 or 1.0). b: Markers of myocardial injury (e.g. elevated cardiac troponin I or -T), or of heart failure biomarkers (BNP or NT-BNP); c: Either one of imaging or laboratory examination is positive.
