# Supplementary material for: Oxygenation index and NT-proBNP as predictors of pulmonary hypertension and ventilation/perfusion mismatch in acute pulmonary embolism
Source: Front Cardiovasc Med. 2023 Feb 6;10:1090805. doi: 10.3389/fcvm.2023.1090805 (PMC9940751; doi:10.3389/fcvm.2023.1090805)
Supplement: Supplementary file 2 [file Table_2.docx]

STable 2. Differences between couples of groups

|  |  | **High-risk** | **Intermediate-risk** | **Low-risk** |
| --- | --- | --- | --- | --- |
| **Male gender (%)** | **High-risk** | / | 0.241 | 0.055 |
|  | **Intermediate-risk** | / | / | 0.280 |
|  | **Low-risk** | / | / | / |
| **Age (years)** | **High-risk** | / | 0.311 | 0.315 |
|  | **Intermediate-risk** | / | / | 0.991 |
|  | **High-risk** | / | / | / |
| **D-Dimer (mg/L),** | **High-risk** | / | 0.625 | <0.001 |
|  | **Intermediate-risk** | / | / | <0.001 |
|  | **High-risk** | / | / | / |
| **TNI (ng/ml),** | **High-risk** | / | 0.232 | <0.001 |
|  | **Intermediate-risk** | / | / | <0.001 |
|  | **High-risk** | / | / | / |
| **NT-proBNP (pg/ml)** | **High-risk** | / | 0.783 | <0.001 |
|  | **Intermediate-risk** | / | / | <0.001 |
|  | **High-risk** | / | / | / |
| **PO2 (kpa)** | **High-risk** | / | 0.041 | <0.001 |
|  | **Intermediate-risk** | / | / | 0.017 |
|  | **High-risk** | / | / | / |
| **OI (mmHg)** | **High-risk** | / | <0.001 | <0.001 |
|  | **Intermediate-risk** | / | / | <0.001 |
|  | **High-risk** | / | / | / |
| **PAP (mmHg)** | **High-risk** | / | 0.609 | <0.001 |
|  | **Intermediate-risk** | / | / | <0.001 |
|  | **High-risk** | / | / | / |
| **Number of V/Q mismatched lung segments (n)** | **High-risk** | / | <0.001 | <0.001 |
|  | **Intermediate-risk** | / | / | 0.263 |
|  | **High-risk** | / | / | / |
| **PVO (%)** | **High-risk** | / | <0.001 | <0.001 |
|  | **Intermediate-risk** | / | / | <0.001 |
|  | **High-risk** | / | / | / |
| **Hospitalization days (range)** | **High-risk** | / | 0.589 | 0.036 |
|  | **Intermediate-risk** | / | / | 0.026 |
|  | **High-risk** | / | / | / |

NOTE: Data are presented as mean (SD), median (IQR), n (%). TNI: troponin I; NT-proBNP: N-terminal pro-brain natriuretic peptide; PO2: partial oxygen pressure; V/Q scan: ventilation-perfusion lung scintigraphy; PAP: pulmonary artery pressure; PVO: pulmonary vascular obstruction; OI: oxygenation index.
