# Supplementary material for: Oxygenation index and NT-proBNP as predictors of pulmonary hypertension and ventilation/perfusion mismatch in acute pulmonary embolism
Source: Front Cardiovasc Med. 2023 Feb 6;10:1090805. doi: 10.3389/fcvm.2023.1090805 (PMC9940751; doi:10.3389/fcvm.2023.1090805)
Supplement: Supplementary file 3 [file Table_3.docx]

STable 3: Influencing factors by linear regression analysis in different risk groups

|  |  | **Univariate linear regression** | | **Multivariate linear regression** | |
| --- | --- | --- | --- | --- | --- |
|  |  | **βCoefficient**  **(95% CI)** | **P** | **βCoefficient**  **(95% CI)** | **P** |
| **Factor for PAP** | | | | | |
| High-risk | NT-proBNP(pg/ml) | 0.003  (0.001,0.006) | 0.011 | 0.003  (0.001,0.006) | 0.010 |
|  | OI (mmHg) | –0.018  (–0.075, 0.039) | 0.519 | –0.010  (–0.063, 0.043) | 0.751 |
|  | TNI (ng/ml) | –4.060  (–35.932, 27.812) | 0.796 | –12.899  (–43.486, 17.689) | 0.395 |
| Intermediate-risk | NT-proBNP(pg/ml) | 0.000  (–0.001,0.002) | 0.180 | 0.001  (0.000,0.002) | 0.228 |
|  | OI (mmHg) | –0.015  (–0.036, 0.006) | 0.161 | –0.014  (–0.035, 0.007) | 0.199 |
|  | TNI (ng/ml) | 0.750  (–3.889, 5.389) | 0.749 | –0.221  (–4.965, 4.522) | 0.926 |
| Low-risk | NT-proBNP(pg/ml) | 0.007  (–0.018,0.004) | 0.145 | 0.004  (–0.006,0.014) | 0.466 |
|  | OI (mmHg) | –0.007  (–0.018, 0.004) | 0.186 | –0.007  (–0.018, 0.003) | 0.179 |
|  | TNI (ng/ml) | 126.951  (–9.274, 263.176) | 0.067 | 108.915  (–38.793, 265.263) | 0.146 |
| **Factor for PVO** | | | | | |
| High-risk | NT-proBNP(pg/ml) | 0.000  (–0.001, 0.001) | 0.317 | 0.000  (–0.002,0.001) | 0.342 |
|  | OI (mmHg) | –0.005  (–0.031, 0.022) | 0.729 | –0.005  (–0.033, 0.023) | 0.707 |
|  | TNI (ng/ml) | –2.373  (–17.261, 12.515) | 0.747 | –0.017  (–-16.037, 16.002) | 0.998 |
| Intermediate-risk | NT-proBNP(pg/ml) | 0.000  (–0.002, 0.001) | 0.487 | 0.000  (–0.002,0.001) | 0.318 |
|  | OI (mmHg) | 0.004  (–0.022, 0.029) | 0.778 | 0.005  (–0.021, 0.030) | 0.716 |
|  | TNI (ng/ml) | 3.826  (–1.630, 9.283) | 0.167 | 4.567  (–-1.076, 10.211) | 0.111 |
| Low-risk | NT-proBNP(pg/ml) | 0.023  (–0.002, 0.048) | 0.065 | 0.026  (0.000,0.053) | 0.058 |
|  | OI (mmHg) | –0.026  (–0.054, 0.002) | 0.072 | –0.024  (–0.052, 0.004) | 0.094 |
|  | TNI (ng/ml) | –2.876  (–373.209, 367.457) | 0.988 | –140.075  (–-532.316, 252.166) | 0.480 |

NOTE: TNI: troponin I; NT-proBNP: N-terminal pro-brain natriuretic peptide; PAP: pulmonary artery pressure; PVO: pulmonary vascular obstruction, OI: oxygenation index.
